# Surgical Management of Gastroesophageal Reflux in Neurologically Impaired Children: Fundoplication vs. Total Esophagogastric Dissociation

**DOI:** 10.3390/jcm14041058

**Published:** 2025-02-07

**Authors:** Marco Di Mitri, Marzia Vastano, Annalisa Di Carmine, Enrico Oriani, Eduje Thomas, Cristian Bisanti, Simone D’Antonio, Vincenzo Davide Catania, Edoardo Collautti, Tommaso Gargano, Mario Lima

**Affiliations:** Pediatric Surgery Department, IRCCS Sant’Orsola, Alma Mater Studiorum University of Bologna, 40126 Bologna, Italy; marcodimitri14@gmail.com (M.D.M.); marzia.vastano@icloud.com (M.V.); annalisa.dicarmine@gmail.com (A.D.C.); enrico.oriani@studio.unibo.it (E.O.); edu.thomas92@gmail.com (E.T.); bisanticristian96@gmail.com (C.B.); simone.dantonio@aosp.bo.it (S.D.); vdcatania1985@gmail.com (V.D.C.); edocolla.ec@gmail.com (E.C.); tommaso.gargano2@unibo.it (T.G.)

**Keywords:** gastro-esophageal reflux, fundoplication, total esophagogastric dissociation, neurologically impaired children

## Abstract

**Background:** Gastroesophageal reflux disease (GERD) is a prevalent and severe condition in neurologically impaired (NI) children, often requiring surgical intervention. This study evaluates the outcomes of two surgical techniques, fundoplication and total esophagogastric dissociation (EGD), in managing GERD in this vulnerable population. **Methods:** We conducted a retrospective analysis of 56 NI children who underwent surgery for GERD at our institution from 2012 to 2023. Outcomes assessed included post-operative weight gain, pneumonia rates, hospitalization duration, and complications. **Results:** Of the cohort, 39 patients underwent fundoplication and 17 underwent EGD. Both groups experienced significant weight gain post-operatively, with comparable rates between procedures. Fundoplication was associated with shorter hospitalization (16 ± 10 days vs. 35 ± 16 days, *p* < 0.001) and earlier resumption of enteral feeding (5.5 ± 2 days vs. 10.2 ± 3 days). EGD significantly reduced pneumonia rates (70.59% to 17.65%, *p* = 0.006) compared to fundoplication (58.97% to 41.03%, *p* = 0.174). Early complication rates were higher in the EGD group (41% vs. 23%), but long-term GERD-related hospitalizations were fewer (0.8 ± 1/year vs. 3 ± 2/year, *p* = 0.003). **Conclusions:** Fundoplication offers shorter recovery times and lower early complication rates, making it suitable for many patients. However, EGD may be preferable for those with severe, refractory GERD, given its superior long-term outcomes, particularly in reducing pneumonia and hospitalizations. Prospective studies are needed to confirm these findings and refine surgical indications in NI children.

## 1. Introduction

Gastroesophageal reflux disease (GERD) is a common and significant issue among children with neurological impairment (NI), such as cerebral palsy, congenital brain anomalies, and other neurodevelopmental disorders [1]. These children are particularly vulnerable to GERD due to their compromised neuromuscular coordination, chronic immobility, and associated feeding difficulties; because of these pathological issues, the literature reported a high prevalence of GERD in NI patients with an incidence of 70% [1,2,3]. GERD occurs when the contents of the stomach, including acid and partially digested food, flow back into the esophagus, causing discomfort and potentially leading to more serious complications, such as aspiration pneumonia, failure to thrive, and esophagitis. In NI, GERD is often more severe, persistent, and resistant to medical treatment, leading to considerable morbidity and a significant reduction in quality of life [4,5].

The management of GERD in NI children poses a complex clinical challenge. Conservative treatments, such as lifestyle modifications, thickened feeds, and pharmacological therapies, are often ineffective in this population, largely due to the underlying neurological issues that can inflame reflux. While proton pump inhibitors and H2 receptor antagonists can provide temporary relief, they fail to address the mechanical issues, often resulting from poor motility and abnormal functioning of the lower esophageal sphincter [6]. For these reasons, surgical intervention is frequently considered the definitive treatment for GERD in NI children, especially in cases where medical management fails or complications arise, such as recurrent aspirations or poor weight gain [7].

Several surgical options are available for the management of GERD, with the two most frequently employed procedures being fundoplication and esophagogastric disconnection (EGD). Both procedures aim to prevent the reflux of gastric contents into the esophagus, but they differ significantly in their techniques, goals, and potential outcomes. Fundoplication, first described by Dr. Rudolph Nissen in the 1950s, involves wrapping the upper part of the stomach (the fundus) around the lower esophagus to reinforce the lower esophageal sphincter and prevent reflux [8]. This procedure has long been regarded the standard surgical approach for GERD in both children and adults, and several variations, including the Nissen, Toupet, and Thal fundoplications, have been developed to tailor the procedure to the needs of different patient populations [9].

In NI patients, fundoplication has shown mixed results. While it can be effective in alleviating GER symptoms and preventing aspiration in certain cases, many studies report high rates of recurrence and complications, including dysphagia (difficulty in swallowing), gas-bloat syndrome, and the necessity for revision surgery. Additionally, the underlying neurological impairment in these patients frequently leads to poor outcomes following fundoplication, as the procedure does not address the root cause of the motility disorder. This has led some surgeons to explore alternative surgical approaches for managing GERD in this vulnerable population [10].

Esophagogastric dissociation (EGD) is a viable alternative that has gained attention in recent years. EGD is a more radical procedure that entails the division of the esophagus from the stomach, establishing a permanent dissociation between these two structures. This procedure prevents any potential for reflux, as gastric contents can no longer reach the esophagus. To ensure that the patient can still receive adequate nutrition, a gastrostomy tube is placed directly into the stomach or small intestine to allow for enteral feeding. While this procedure is less commonly performed than fundoplication, it has been proposed as a viable option for patients with severe GERD who are not candidates for less invasive procedures or who have failed previous fundoplications [9].

The theoretical advantage of esophagogastric disconnection lies in its ability to completely eliminate reflux, which may reduce the risk of aspiration and other reflux-related complications. However, this procedure is also associated with significant drawbacks, including the permanent nature of the procedure, the need for lifelong enteral feeding, and the risk of long-term nutritional and gastrointestinal complications. Furthermore, the radical nature of the surgery raises concerns about its appropriateness in children, especially in those with complex medical conditions such as NI children [10].

Given the complexities of GERD management in NI children and the lack of consensus on the optimal surgical approach, there is a clear need for studies that compare the clinical outcomes of fundoplication and esophagogastric dissociation in this population. The existing literature on the subject is limited and frequently presents conflicting results, with some studies suggesting that fundoplication is associated with fewer complications and improved quality of life, while others indicate that EGD may be more effective in preventing life-threatening complications, such as aspiration pneumonia. In addition, most studies have small sample sizes and are retrospective in nature, limiting their generalizability and clinical relevance [11,12].

Surgery for GERD’s treatment can be performed using either an open technique or a minimally invasive approach. The table below outlines the advantages and disadvantages of both surgical methods (Table 1).

In light of these gaps in the literature, the primary objective of our study was to identify which surgical technique—fundoplication or esophagogastric dissociation—produced the best clinical outcomes in NI patients affected by GERD. Specifically, we sought to evaluate the efficacy of each procedure in reducing GERD symptoms, preventing aspiration, improving nutritional status, and minimizing the need for additional surgical interventions. We aimed to assess the safety profiles of the two procedures as well, including the rates of post-operative complications and long-term morbidity. By elucidating the relative benefits and risks associated with fundoplication and esophagogastric dissociation, we aim to enhance the understanding of GER management in NI children and improve the overall quality of care for this challenging patient group.

## 2. Methods

A retrospective observational single-center study was conducted in our Department of Pediatric Surgery, IRCCS Sant’Orsola-Malpighi University Hospital of Bologna, following Ethical Committee approval (CHPED-21-DEG). Clinical records were retrospectively analyzed to identify all NI children who underwent surgery for GERD in our department between January 2012 and January 2023. Patients with less than one year of follow-up were excluded from the study to ensure consistency in post-operative outcome assessment (Table 2).

In our center, EGD was performed using the Bianchi technique, while the Nissen technique was used for the fundoplication surgery. Concomitant medical management played a pivotal role in optimizing outcomes for children undergoing surgical interventions for GERD, particularly those with neurological impairments. In our study, we emphasized the use of additional pharmacological therapies to manage symptoms and prevent complications. Proton pump inhibitors (PPIs) were routinely prescribed to reduce gastric acid production and promote mucosal healing in cases of gastroesophageal reflux. Additionally, prokinetic agents were utilized to enhance gastric motility and reduce the risk of delayed gastric emptying, which is a common issue in this population. In patients with significant muscle tension or spasticity, muscle relaxants were considered to alleviate esophageal dysmotility and improve swallowing coordination. Pre-operative management included addressing active reflux esophagitis with a combination of PPIs and dietary modifications to minimize inflammation at the time of surgery. Post-operatively, the continuation of medical therapy was tailored to each patient’s clinical response and the severity of their underlying condition. This integrated approach ensured that surgical treatment was complemented by medical management to enhance both short- and long-term outcomes. In NI children, a common comorbidity is scoliosis, which can complicate both medical and surgical management. Our approach involved a multidisciplinary strategy to optimize pre-operative, intra-operative, and post-operative care. Pre-operatively, all children with scoliosis underwent thorough evaluation by an orthopedic specialist to assess the severity of spinal curvature and its impact on thoracic and abdominal anatomy. Particular attention was given to the degree of spinal deformity and its potential to affect positioning during surgery, as well as its influence on respiratory and gastrointestinal function. Intra-operatively, adjustments were made to surgical positioning to accommodate spinal curvature and ensure optimal access to the surgical field. For children with severe scoliosis, specialized padding and supports were used to stabilize their posture and minimize the risk of additional complications. Post-operatively, intensive monitoring and coordinated follow-ups with orthopedic care were provided to manage any scoliosis-related issues that could influence recovery or long-term outcomes.

### 2.1. Surgical Technique

**Figure 1:**
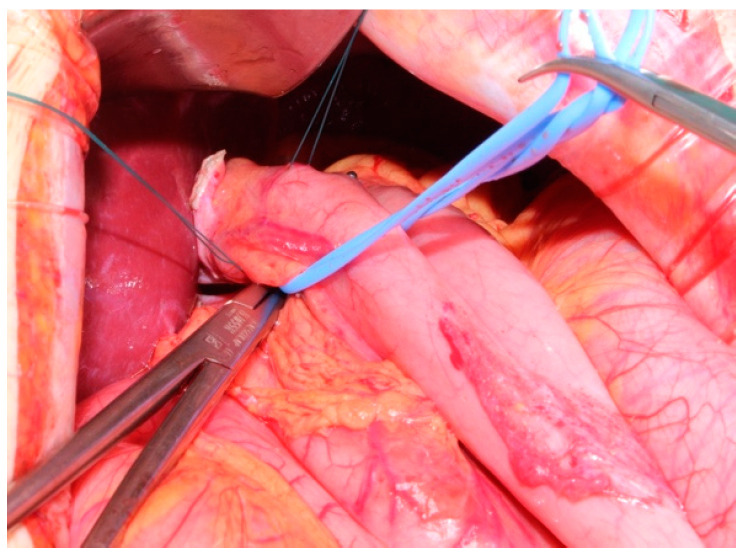
Total esophagogastric dissociation. Transection of esophagogastric junction.

**Figure 2:**
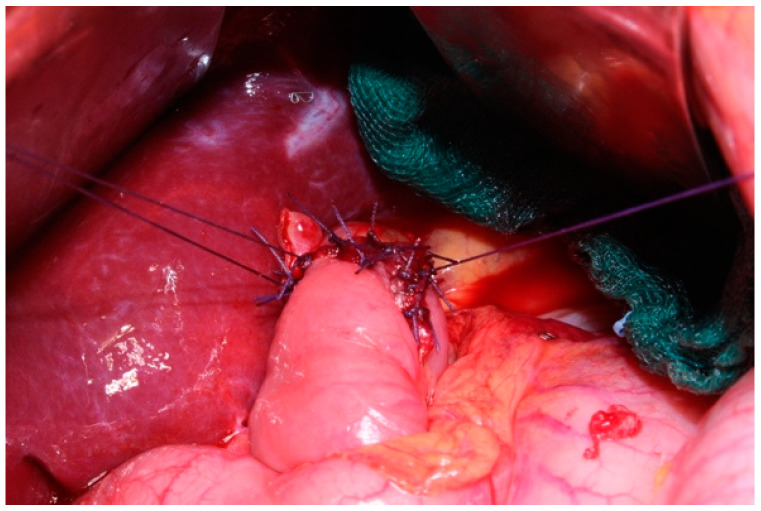
Total esophagogastric dissociation. Anastomosis between esophagus and jejunal loop.

**Figure 3:**
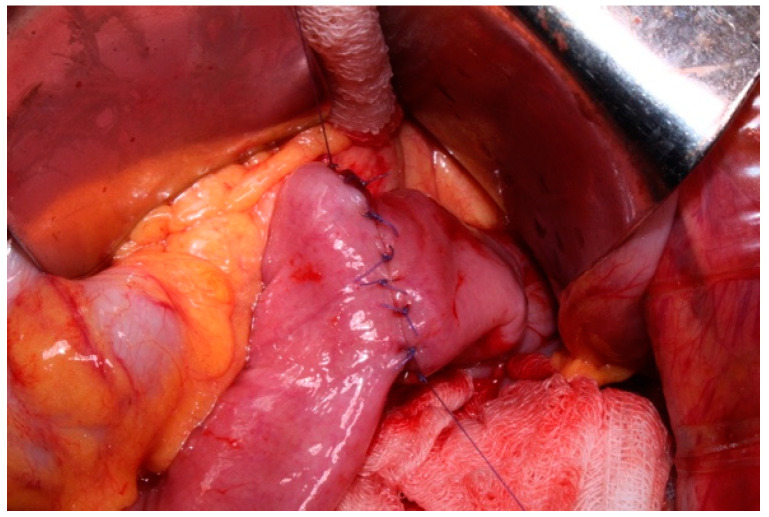
Total esophagogastric dissociation. End-to-side jejuno-jejuno anastomosis.

In our center EGD is performed using the Bianchi technique, which consists of the insolation of the esophagogastric junction and its transection (Figure 1), along with the mobilization of a jejunal loop with a vascular pedicle without tension. The loop is brought through the transverse mesocolon and an anastomosis with the proximal esophagus is performed (Figure 2). There is no standardized length for the Roux loop, but generally, it measures around 20–30 cm from the esophagojejunal anastomosis. Then, an end-to-side jejuno-jejunal anastomosis is performed to establish bowel continuity (Figure 3) [13].

### 2.2. Statistics

Data were analyzed using descriptive and inferential statistics to compare the clinical outcomes of fundoplication and EGD in NI children with GERD. Continuous variables, such as patient age, weight gain, and time of hospitalization, are presented as means with standard deviations (Mean ± SD), and categorical variables are summarized as frequencies and percentages.

Comparisons between the two surgical groups were performed using the independent sample *t*-test for continuous variables (weight gain, time to resume feeding, length of hospital stay) to assess the mean differences between the groups. For categorical variables, including post-operative complications and pneumonia rates, the Chi-square test or Fisher’s exact test was employed, as appropriate, to evaluate differences in proportions between the groups.

Statistical significance was set at a *p*-value of < 0.05.

## 3. Results

Between January 2012 and January 2023, a total of 56 NI children underwent surgical intervention for GERD at our Pediatric Surgery Department. Of all patients, 39 children (group 1), 16 (41%) females and 23 (59%) males, underwent fundoplication, and 17 children (group 2), 9 (53%) females and 8 (47%) males, underwent EGD. The mean age at surgery was 5.6 years (range = 0.5–30 years) for group 1 and 3.6 years (range = 1–27 years) for group 2. All patients had at least one year of follow-up to ensure consistent post-operative outcome assessment.

### 3.1. Group 1—NI Children Who Underwent Fundoplication

The primary neurological conditions affecting the 39 patients who underwent fundoplication and were included in the study are as follows: lissencephaly (2.6%), muscular dystrophy (5.2%), Kleefstra syndrome (2.6%), spastic tetraparesis with epilepsy (26.3%), spastic tetraparesis without epilepsy (10.5%), vanishing white matter leukoencephalopathy (2.6%), Joubert syndrome (2.6%), congenital toxoplasmosis (2.6%), Leigh syndrome (2.6%), Ellis Van Creveld syndrome (2.6%), Pelizaeus-Merzbacher d (2.6%), hypoxic–ischemic encephalopathy (21%), West syndrome (5.2%), cerebellar hypotonic tetraparesis (2.6%), Rubinstein-Taybi syndrome (2.6%), mitochondrial encephalopathy (2.6%), and polymicrogyria (2.6%).

Of the 39 patients who underwent fundoplication, 20 (51%) underwent the Nissen technique, 17 (44%) underwent the Dor technique, and 2 (5%) underwent the Nissen–Rossetti technique. Minimally invasive surgery was used for 30 (77%) patients as follows: 28 (72%) patients underwent a laparoscopic approach, and 2 (5%) patients underwent a robotic approach. For the remaining 9 (23%) patients, the open approach was preferred. Of the 39 patients who underwent fundoplication, 6 patients required redo fundoplication (15%), and 7 patients subsequently underwent esophagogastric dissociation (18%).

### 3.2. Group 2—NI Children Who Underwent EGD

The underlying conditions affecting patients who underwent EGD and were included in the study are as follows: Warburg syndrome (5.8%), Moebius syndrome (11.7%), hypoxic–ischemic encephalopathy (23.5%), spastic tetraparesis with epilepsy (5.8%), spastic tetraparesis without epilepsy (17.6%), Ellis Van Creveld syndrome (5.8%), epileptic encephalopathy due to PIGN gene mutation (11.7%), Edwards syndrome (Trisomy 18) (5.8%), and cerebellar encephalopathy (5.8%). In all patients, EGD was performed using the Bianchi technique.

### 3.3. Weight

The mean pre-operative weight was 15 ± 9 kg for group 1 and 14 ± 8 kg for group 2. The patients who underwent EGD experienced greater weight gain compared to those who underwent fundoplication, reaching a mean weight at the one-year follow-up of 20 ± 11 kg for group 1 and 19 ± 11 kg for group 2. The weight gain rate in groups 1 and 2 was 35% and 40%, respectively. Regarding the resumption of enteral feeding, patients in group 1 resumed earlier than those in group 2, at 5.5 post-operative days (POD) compared to 10.2 POD, and reached full enteral feeding at 12 POD and 20 POD, respectively (Table 3).

### 3.4. Time of Hospitalization

Analyzing the time of hospitalization (TOH), we detected a longer TOH for group 2 compared with group 1 (*p* < 0.001), both for hospitalization in intensive care and hospitalization in our department (Table 4).

### 3.5. Post-Operative Complications

We analyzed the early (<30 days) and late (>30 days) complications after surgery in both group 1 and group 2. The most common complications were respiratory diseases, which occurred in 8 patients (20%) in group 1 and 5 patients (29%) in group 2. Others reported complications including surgical site infections and post-operative emesis. The total early complication rate was 23% for group 1 and 41% for group 2. No statistically significant differences were observed when comparing individual complication and the total rate of complications (*p* > 0.05).

The impact of surgery on pneumonia rates was analyzed in both groups. In group 1 (n = 39), the pneumonia rate decreased from 58.97% pre-operatively to 41.03% post-operatively, though this reduction was not statistically significant (*p* = 0.174). In group 2 (n = 17), the pneumonia rate dropped from 70.59% to 17.65% post-operatively, with this reduction being statistically significant (*p* = 0.006). These findings suggest that although both procedures reduced pneumonia rates, only EGD resulted in a significant decrease.

We also included, in the late complication analysis, the rate of post-operative hospitalizations related to GERD. The mean rate of hospitalization was 3 ± 2 in group 1 and 0.8 ± 1 in group 2, showing a statistically significant decrease in the rate of new hospitalizations for group 2 (*p* = 0.003).

## 4. Discussion

GERD is a significant clinical challenge in NI children due to their compromised neuromuscular coordination and feeding difficulties. GERD is highly prevalent in children with neurological impairments, and its association is multifactorial. NIPs often exhibit dysfunctions in swallowing reflexes and esophageal motility, which contribute to the development of GERD. Impaired coordination between the pharyngeal and esophageal phases of swallowing, frequently observed in these patients, leads to prolonged esophageal acid exposure [1]. Furthermore, conditions such as hypotonia and increased intra-abdominal pressure—common in children with severe neurological disorders—can exacerbate the risk of reflux. Studies have shown that esophageal dysmotility, including delayed gastric emptying, further aggravates GERD in this population. Additionally, recurrent episodes of aspiration, a common complication in NIPs, not only worsen respiratory outcomes but also perpetuate the cycle of GERD [1,2]. Recognizing these pathophysiological mechanisms is critical for tailoring effective medical and surgical interventions to treat GERD in neurologically impaired pediatric patients. Despite conservative treatments, surgical intervention frequently becomes necessary for the effective management of GERD [1]. The two most common surgical procedures—fundoplication and EGD—present distinct outcomes and challenges, as highlighted in our study and in the existing literature [14]. Post-operative weight gain is a critical marker of improved nutritional status, especially in NI children who often suffer from feeding difficulties. In our study, both groups showed significant weight gain after surgery, with the fundoplication group reaching a mean weight of 20 ± 11 kg and the EGD group reaching 19 ± 11 kg. The weight gain rate was slightly higher in the EGD group (40%) compared to the fundoplication group (35%), which is consistent with the existing literature indicating improved nutritional outcomes following these procedures. However, EGD patients experienced a delayed resumption of enteral feeding, with a mean of 10.2 days post-operatively compared to 5.5 days for the fundoplication group. Similar results were reported by Gatti et al. in their series [15].

Negri et al. confirmed this finding in their systematic review, concluding that all surviving patients experienced weight gain at follow-up [16].

Complication rates in our study were higher in the EGD group, particularly for early post-operative complications, with 41% of EGD patients experiencing complications compared to 23% of those in the fundoplication group. Respiratory complications were the most common, affecting 20% of fundoplication patients and 29% of EGD patients. This finding is consistent with previous reports detecting higher early complication rates with EGD due to the more invasive nature of the surgery. Despite the increased complication rates in the early post-operative period, long-term outcomes favored EGD, as evidenced by the fewer hospitalizations related to GERD recurrence in the EGD group (0.8 ± 1 per year) compared to the fundoplication group (3 ± 2 per year). This suggests that although EGD is associated with a higher immediate risk, it provides more sustained relief from GERD in the long term.

Our results are supported by the literature, for instance, the reported by Bianchi et al., who highlighted that early complications following EGD, including respiratory distress, occur more frequently in EGD than in fundoplication due to the radical nature of the procedure [13].

Moreover, Danielson et al. reported similar results in their series including 27 patients, where 30% experienced early post-operative complications, and late complications were observed in 41%, with 15% requiring reoperation [17]. Buratti et al. presented similar results in their paper. In their series including seven children, they showed a rate of early complications in 43% of patients, including the necrosis of the Roux-en-Y loop, bilious drainage from the gastrostomy, and post-operative edema of the esophagojejunal anastomosis with obstruction, a case of wound infection and one death from sepsis and respiratory failure [12].

A notable finding in our study was the significant reduction in pneumonia rates observed post-operatively in the EGD group. The pneumonia rate dropped from 70.59% pre-operatively to 17.65% post-operatively (*p* = 0.006), a significant reduction that highlights the procedure’s effectiveness in preventing aspiration-related complications. Fundoplication reduced pneumonia rates too, from 58.97% to 41.03%, though this reduction was not statistically significant (*p* = 0.174). Previous studies have likewise reported that EGD is particularly effective in preventing aspiration pneumonia due to its ability to eliminate reflux of gastric contents. Parente et al. reported a reduction in hospitalizations for pneumonia after EGD [11]. Similar results were presented by Verey et al., who analyzed the outcomes of patients who underwent EGD and concluded that most of the authors would consider EGD as a primary approach in severely NI children due to the lower rate of post-operative pneumonia [18].

Contrasting results were described in the literature about the risk of pneumonia after fundoplication in NI patients. Lee et al., in their series including 342 pediatric patients, showed that no overall improvement in hospitalization for GERD-related pulmonary complications after Nissen fundoplication was observed [19].

Contradictory to these results, Maret-Ouda et al., in their population-based cohort study from Denmark, Finland, Norway, and Sweden including 578 NI children, analyzed the risk of aspiration pneumonia after fundoplication surgery. The authors concluded that fundoplication was associated with a 56% decrease in the overall HR of aspiration pneumonia (HR 0.44, 95% CI 0.27–0.72) [20].

According to these results, a large retrospective cohort study by Srivastava et al., using a sample of 3721 NI children with GERD, showed a reduction in aspiration pneumonia (incidence rate ratio 0.71, 95% CI 0.62–0.81) after fundoplication [21].

An interesting study by Gatti et al. analyzed the rate of aspiration pneumonia, comparing results after fundoplication and EGD. They divided the patients (n = 26) in two groups, showing that the group that underwent EGD resulted in a reduction in respiratory infections and improved quality of life, suggesting choosing EGD as the primary procedure for NI children with refractory GERD [15].

The length of post-operative hospitalization was significantly longer for the EGD group, with a mean total hospitalization time of 35 ± 16 days compared to 16 ± 10 days in the fundoplication group (*p* < 0.001). This longer hospitalization is consistent with the more invasive nature of the EGD procedure, which requires more extensive post-operative care, including longer stays in the intensive care unit. Furthermore, the difference in length of hospitalization could also be attributed to the use of an open surgical technique for EGD, as compared to the minimally invasive approaches commonly used for fundoplication. The potential to reduce post-operative hospitalization through the application of minimally invasive robotic surgery for EGD has been demonstrated by Mattioli et al., suggesting that such advancements could help shorten recovery times and improve overall outcomes for EGD patients [22].

### Limitations of the Study and Future Perspectives

We are aware of the limits of this study. First, this is a retrospective study which may introduce biases related to patient selection, the completeness of medical records, and the accuracy of follow-up data. Second, the heterogeneity of the patient population is another limitation, since the neurological conditions and severity of impairment vary widely within the study groups. Additionally, while the one-year follow-up period is adequate for evaluating short-term complications and initial outcomes, it may not fully capture the long-term recurrence of GERD symptoms or the potential development of late complications.

Future studies should aim to stratify patients by the type and severity of neurological impairment in order to better assess the outcomes in more homogenous subgroups and to evaluate emerging surgical techniques, such as minimally invasive EGD (laparoscopic or robotic) or newer anti-reflux procedures.

## 5. Conclusions

In conclusion, both fundoplication and EGD are viable options for managing GERD in NI children, but they have distinct profiles in terms of complications, recovery time, and long-term efficacy. Fundoplication is associated with shorter hospitalization times and lower early complication rates, making it a good option for many patients. However, EGD may be more appropriate for those with severe, refractory GERD, as it offers better long-term outcomes, particularly in preventing recurrent pneumonia and reducing re-hospitalization rates. Future prospective studies with larger sample sizes are necessary to further clarify the role of each procedure in managing GERD in this vulnerable patient population.

## Figures and Tables

**Table 1:** Advantages and disadvantages of both surgical methods.

**Laparoscopic Approach**	
**Advantages**	**Disadvantages**
Less post-operative pain and faster recovery	Technically demanding, requiring specialized training and equipment
Shorter hospital stay and less healthcare costs	Limited visualization and maneuverability in some complex cases
Less risk of surgical site infections	Not suitable for patients with extensive adhesions or anatomical abnormalities
**Open Surgery**	
**Advantages**	**Disadvantages**
Allows direct visualization and manipulation of the surgical field	Longer recovery time
Suitable for complex cases, including patients with significant intestinal adhesions	Higher rate of surgical site infections and post-operative complications
Lower technical requirements compared to laparoscopic techniques	Longer hospital stays and higher overall healthcare costs

**Table 2:** Patient inclusion and exclusion criteria for the study.

Inclusion Criteria	Exclusion Criteria
NI patients 0–30 years old	Patients with follow-up < 1 year
Patients with follow-up > 1 year	Non-NI children with GERD
NI patients who underwent fundoplication between January 2012 and January 2023	
NI patients who underwent EGD between January 2012 and January 2023	

**Table 3:** Comparison of pre- and post-operative weight, weight gain rates, and post-operative feeding parameters between group 1 and group 2 gain and post-operative feeding.

		Group 1	Group 2
**Pre-operative weight**	Mean ± DS	15 ± 9	14 ± 8
	Range	3–40	3–29
**Post-operative weight**	Mean ± DS	20 ± 11	19 ± 11
	Range	6–55	6–50
**Weight gain rate**	-	35%	40%
**Post-operative feeding—start (gpo)**	Mean ± DS	5.5 ± 2	10 ± 3
**Post-operative feeding—full (gpo)**	Mean ± DS	12 ± 4	20 ± 7

**Table 4:** Comparison of hospitalization duration (intensive care, Pediatric Surgery Department, and total stay) Between Group 1 and Group 2.

Hospitalization		Group 1	Group 2	*p* Value
**Intensive care**	Mean ± DS	2.8 ± 4	5.7 ± 3	<0.001
**Pediatric Surgery Department**	Mean ± DS	13.5 ± 8	30 ± 15	<0.001
**Total**	Mean ± DS	16 ± 10	35 ± 16	<0.001

## Data Availability

The original contributions presented in this study are included in the article. Further inquiries can be directed to the corresponding author(s).

## Abbreviations

**NI**	Neurologically impaired
**GERD**	Gastroesophageal reflux disease
**EGD**	Total esophagogastric dissociation
**TOH**	Time of hospitalization
